# Promising Biomarker Candidates for Cardioembolic Stroke Etiology. A Brief Narrative Review and Current Opinion

**DOI:** 10.3389/fneur.2021.624930

**Published:** 2021-02-25

**Authors:** Arnold Markus, Schütz Valerie, Katan Mira

**Affiliations:** Department of Neurology, University Hospital of Zurich, Zurich, Switzerland

**Keywords:** biomarker, cardioembolic stroke, stroke etiology, ischemic stroke, secondary prevention

## Abstract

Determining the cause of stroke is considered one of the main objectives in evaluating a stroke patient in clinical practice. However, ischemic stroke is a heterogeneous disorder and numerous underlying disorders are implicated in its pathogenesis. Although progress has been made in identifying individual stroke etiology, in many cases underlying mechanisms still remain elusive. Since secondary prevention strategies are tailored toward individual stroke mechanisms, patients whose stroke etiology is unknown may not receive optimal preventive treatment. Cardioembolic stroke is commonly defined as cerebral vessel occlusion by distant embolization arising from thrombus formation in the heart. It accounts for the main proportion of ischemic strokes, and its share to stroke etiology is likely to rise even further in future decades. However, it can be challenging to distinguish cardioembolism from other possible etiologies. As personalized medicine advances, stroke researchers' focus is increasingly drawn to etiology-associated biomarkers. They can provide deeper insight regarding specific stroke mechanisms and can help to unravel previously undetected pathologies. Furthermore, etiology-associated biomarkers could play an important role in guiding future stroke prevention strategies. To achieve this, broad validation of promising candidate biomarkers as well as their implementation in well-designed randomized clinical trials is necessary. This review focuses on the most-promising candidates for diagnosis of cardioembolic stroke. It discusses existing evidence for possible clinical applications of these biomarkers, addresses current challenges, and outlines future perspectives.

## Introduction

Due to an aging world population and a global shift from communicable to non-communicable disease, lifetime risk for stroke and the absolute numbers of stroke survivors are increasing all over the world (1, 2). Ischemic stroke caused by an acute arterial occlusion is responsible for the majority of stroke events. In the acute phase of ischemic stroke, treatment strategies are focusing on the rapid reperfusion of affected tissue and the prevention of acute complications. Yet, patients who survive initial ischemic stroke share a high risk for disease recurrence. It is estimated that within five years at least one out of six stroke survivors is affected by a second event, underscoring the importance of optimal secondary prevention (3). Preventive strategies generally recommended for all patients include common elements of cardiovascular risk factor management like blood pressure control, cholesterol reduction, treatment of diabetes mellitus, and antiplatelet medication. However, given the heterogeneity of possible underlying stroke mechanisms, specific preventive interventions such as oral anticoagulation in patients with atrial fibrillation (AF) or carotid endarterectomy for symptomatic carotid artery stenosis are tailored toward an individual etiology. Thus, etiological classification is crucial for secondary prevention.

## Cardioembolic Stroke Etiology

Stroke caused by cardioembolism (CE) accounts for ~20 to 30% of ischemic strokes with higher incidence in older patients (4). Cardiac embolism is known to cause more severe strokes than other etiologies, and patients with CE strokes share high rates of both early and long-term recurrence (5, 6). Moreover, secular trends indicate that cardiac embolism accounts for an increasing share of strokes in high-income countries as the population ages and treatment of cardiovascular risk factors, especially hypertension and dyslipidemia leading to large-vessel atherosclerosis, improves with socioeconomic progress (7, 8). It is therefore estimated that the absolute numbers of CE strokes will continue to rise during the next decades (9).

AF represents the main identifiable risk factor for CE strokes, and treatment with oral anticoagulation can prevent up to 70% of recurring events in those patients. Less commonly identified causes of CE stroke include severe systolic heart failure, patent foramen ovale (PFO), prosthetic heart valves, recent myocardial infarction, intracardial masses, and endocarditis. For those, different secondary prevention strategies apply, but levels of evidence for optimal preventive strategies in these cases vary greatly [reviewed by Kamel et al. (4)]. Additionally, recent evidence indicates that the relationship between AF and CE stroke may be more complex than assumed. Under the current pathophysiological paradigm, blood stasis caused by arrhythmia leads to the formation of blood clots primarily in the left atrium (LA) from where they can embolize and cause distant brain-vessel occlusion. However, this paradigm is challenged by several findings: There was no clear temporal relationship between episodes of AF and occurrence of stroke in the ASSERT (10) and TRENDS (11) trials, rhythm control strategies were not able to lower stroke risk (12), and the number of cryptogenic stroke patients with newly diagnosed AF after extensive cardiac rhythm monitoring was unexpectedly low (13). Furthermore, it is well recognized that AF by itself is unlikely to be the sole cause for LA thrombogenesis, as the risk for stroke in AF patients is also highly influenced by the presence of common vascular risk factors (14). As a result, the concept of an underlying atrial myopathy as a possible source of cardiac embolism emerged. A diseased LA is histologically and functionally characterized by inflammation, endothelial dysfunction, fibrosis, contractile dysfunction, or structural derangement (15). These risk factors significantly contribute to a prothrombogenic microenvironment, favoring the possibility of thrombus formation and embolization. Therefore, stroke might occur even prior to manifestation of AF, and administration of anticoagulants could be beneficial at this stage (16). Yet, no clear definition of atrial myopathy exists and it remains to be determined how to correctly select patients at high risk for future CE stroke that may benefit from early systemic anticoagulation.

## Limitation of Current Stroke Classification Systems

To date, there is no gold standard for the determination of CE stroke etiology. The identification of a high-risk cardiac source of embolism in the absence of significant arterial disease therefore remains the cornerstone of diagnosis. Attempting to standardize categorization, several classification systems are in use. The most widely accepted tool remains the Trial of Org 10172 in the Acute Stroke Treatment (TOAST) classification system developed in the 1990s (17). It uses diagnostic information from clinical workup to categorize stroke into five etiological categories: (1) large-vessel atherosclerosis, (2) cardioembolism, (3) small vessel disease, (4) other identifiable causes, and (5) stroke of “undetermined” etiology. The latest of the categories is considered the most heterogeneous group as it includes not only cases with lack of evidence for any predefined cause but also cases with incomplete evaluation and evidence for competing plausible causes. The TOAST classification has only moderate inter-examiner reliability (18, 19). Further, as etiological stroke evaluation advances by an increasing use of vascular imaging and long-term cardiac rhythm monitoring, the share of patients with a combination of vascular, cardiac, and other abnormal findings continually rise, inflating the proportion of “undertermined” etiologies by competing evidence. Therefore, up to 30% of strokes must be classified as “of undetermined source” (6) according to the TOAST classification. In an attempt to overcome these problems, a more sophisticated extension of the TOAST classification, the Causative Classification of Stroke (CCS), was developed. It incorporates levels of confidence for specific etiologies to successfully minimize classification of “undetermined etiology” (20, 21).

However, a different problem persists: stroke etiology can change over time. Even if the most likely cause of a stroke is classified non-cardioembolic, patients can still be affected by a CE stroke in the future. This is especially relevant, since the most common stroke mechanisms share common vascular risk factors by various extents. Therefore, adequately estimating the risk for a future stroke event and its etiology is equally important to correctly identify stroke mechanisms, especially for the long-term prophylaxis. Despite being more complicated and less commonly used in clinical practice, the 2013 revised phenotypic ASCOD classification system resembles this medical reasoning better (22). The ASCOD classification system describes all current pathologies that could potentially lead to an ischemic stroke in a given patient. It therefore captures the overlap between possible etiologies and weights the potential causal relationship in regard to the preceding stroke event (23). It does not determine a final stroke etiology *per se* but appreciates future stroke risk while inter-examiner reliability remains high (24, 25). Unfortunately, the ASCOD classification does not serve well as a tool for clinical decision making, due to its more descriptive character. Several other classification systems exist [ASCO, SPARKLE, CISS, KOREAN-TOAST; reviewed by Radu et al. (26)]. Occasional clinical use and lack of supporting data impede in-depth evaluation.

In addition to the aforementioned classification systems, the concept of embolic stroke of undetermined etiology (ESUS) was developed in 2014. ESUS defines a subset of patients with cryptogenic stroke with a non-lacunar stroke based on infarct topography and sufficient diagnostic workup to exclude the presence of a high-risk cardioembolic source or occlusive atherosclerosis (27). The concept was specifically designed to meet the operational criteria necessary for the conduct of randomized secondary prevention trials. However, after the failure of two large clinical trials in demonstrating any benefit of oral anticoagulation in ESUS patients (28, 29), the practical usefulness of the concept has recently been called into question.

## Biomarkers of Cardioembolic Stroke Etiology

Biomarkers of CE-stroke etiology include parameters from cerebral imaging, electrocardiography, echocardiography, advanced cardiac imaging, and blood-based markers. To date, possible clinical applications are mainly risk stratification and acceleration of etiological classification, consequently accelerating optimal treatment. In the presence of competing evidence, biomarkers could increase the level of certainty for a causal relationship of a specific etiology. In cryptogenic strokes (with no evidence for a specific etiology), biomarkers could justify a more extensive workup [e.g., by selecting patients for prolonged cardiac monitoring (PCM)]. Most importantly, some biomarkers have been implemented in the design of randomized secondary prevention trials aiming to characterize high-risk subpopulations for CE stroke (e.g., patients with presumed atrial myopathy). Consequently, biomarkers have the potential to support decision making in future secondary stroke prevention. Table 1 gives an overview on potential biomarkers for CE stroke etiology.

**Table 1:** Summary of potential cardioembolic stroke biomarkers and levels of evidence.

**Nature**	**Biomarker**	**Level of evidence**	**Possible clinical application**
Circulating proteins	**Markers of myocardial stress**		
	NT-proBNP/BNP	Individual patient pooled data and literature-based meta-analysis (30, 31)	Identification of patients that may benefit from anticoagulation in the absence of AF (32); Selection of patients for PCM (33).
	MR-proANP/ANP	Cohort studies (34, 35)	
	Cardiac troponin	Cohort studies (36–38)	Selection of patients for further echocardiography evaluation (39, 40)
	CK-MB	Cohort studies (41)	
	**Markers of inflammation**		
	CRP	Literature-based meta-analysis (42)	
	Interleukin-6	Literature-based meta-analysis (42)	
	Interleukin-1β	Cohort studies (43, 44)	
	Tumor necrosis factor-α	Cohort studies (43, 44)	
	**Markers of coagulation**		
	D-Dimer	Cohort studies (45–50)	Selection of patients to screen for occult cancer (51)
	Fibrin monomer complex	Single cohort study (46)	
	Soluble fibrin	Single cohort study (46)	
	Fibrin degradation products (FDPs)	Single cohort study (46)	
	Soluble thrombomodulin	Single cohort study (52)	
	**Markers of endothelian dysfunction**		
	sRAGE	Single cohort study (45)	
Genomic markers	RNA panels^+^	Single cohort studies (53, 54)	
Metabolites	Plasma metabolites^*^	Single cohort studies (55, 56)	
Findings from Echocardiography Examinations	P-wave terminal force in lead V1 (PTFV1)	Indirect evidence (57, 58)	
	Intra-atrial block (IAB)	Indirect evidence (59)	
	Excessive supraventricular ectopic activity (ESVEA)	Indirect evidence (60)	
Findings from electrocardiography examinations	LA- volume index/LA diameter	Cohort studies (61–63)	Identification of patients that may benefit from anticoagulation in the absence of AF (64); Selection of patients for PCM (65)
	LA strain	Indirect evidence (66)	
Advanced cardiac imaging	Delayed-enhancement cardiac MRI	Indirect evidence (67)	

+ 40 gene and 23 gene panel;

* *free fatty acids; tricarboxylic acid metabolites-succinate, α-ketoglutarate, malate*.

*NT-proBNP, N-terminal pro-B-type natriuretic peptide; BNP, B-type natriuretic peptide; MR-proANP, Midregional pro-atrial natriuretic peptide; CRP, C-reactive protein; LA, left atrium; PCM, prolonged cardiac monitoring; sRAGE, soluble receptor for advanced glycation end products*.

## Blood-Based Biomarkers

Blood-based biomarkers share advantages over other types of biomarkers: They are rapidly measurable, they are cheaper compared to imaging or hands-on examinations, and—if possible confounders and demographic adjusted cutoffs are established—their interpretation is examiner-independent and does not require specific training. To date, blood-based biomarkers are not routinely measured in stroke care and due to a substantial overlap in the pathophysiology of possible stroke mechanisms and shared risk factors, yet no single marker can perfectly distinguish between CE stroke and other etiologies. However, they can still add valuable information for both clinicians and researchers.

## Blood-Based Biomarkers of Myocardial Stress

Blood biomarkers of myocardial stress may add information on subclinical cardiac pathologies, e.g., the presence of underlying atrial myopathy, systolic heart failure, or subclinical AF.

Natriuretic peptides, namely, atrial natriuretic peptide (ANP) and brain natriuretic peptide (BNP) as well as their cleaved by-products MRproANP and NTproBNP, belong to the most widely studied blood biomarkers to distinguish CE from non-CE stroke. For BNP and NT-proBNP, individual patient-pooled data meta-analysis (including data from 23 studies, 2834 patients) (30) as well as literature-based meta-analysis (including data from 16 studies, 2,958 patients) (31) provides compelling evidence for a consistent association with CE stroke etiology. This association was shown to be independent of demographic variables and stroke severity, which is important due to the association of CE etiology with more severe strokes. While less extensively studied, several studies evaluating the role of MR-proANP as a biomarker of CE stroke suggest at least similar associations (34, 35). MR-proANp was further shown to be associated with incidence of CE stroke but not with incidence LAA or SVD stroke (68). For ANP, only preliminary evidence exists. Having an ultra-short half-life, its usefulness in clinical practice is questionable (69). Additionally, for both NT-proBNP and MR-proBNP, it has been shown that the association with CE-stroke etiology remains stable within the acute phase of stroke (70, 71). This is relevant because time-points of measurement can substantially influence the diagnostic utility of a biomarker, especially in hyperacute diseases such as ischemic stroke. While the evidence from individual studies and meta-analysis strongly support the role of natriuretic peptide analysis for etiological stroke classification, there is no universal agreement on optimal discriminatory cutoffs or the preferenced marker for clinical use. Meta-analysis of studies comparing NT-proBNP vs. BNP showed closely equivalent overall diagnostic accuracies (72). In one study that directly compared BNP and NT-proBNP in cryptogenic stroke patients, a higher specificity of BNP for the detection of AF was demonstrated (73). A direct comparison between brain natriuretic peptides and MRproANP is however still lacking.

In regard to a possible clinical application, indirect evidence supporting a role of natriuretic peptides in the selection of patients for oral anticoagulation arose from *post hoc* analysis of the WARSS trial. This was a randomized trial comparing warfarin and aspirin for the prevention of recurrent stroke in patients with presumed non-CE stroke (74). Among 1028 patients, those with plasma NT-proBNP concentrations above the 95th percentile (>750 pg/mL) had a significant reduction in the composite endpoint of stroke or death when being anti-coagulated with warfarin, compared to patients with NT-proBNP levels ≤750 pg/mL (32). These findings suggest that elevated NT-proBNP concentrations may identify a subgroup of patients with occult cardioembolic stroke mechanism (e.g., undiagnosed AF, atrial myopathy, or subclinical systolic heart failure), who could benefit from oral anticoagulation.

More recently, the investigators of the Find-AF_RANDOMIZED_ trial showed that in patients over 60 years BNP levels can help to identify individuals in whom PCM is particularly efficacious to detect previously undiagnosed AF (33). These results support a clinical application of natriuretic peptides in risk stratification for PCM.

Evidence for the role of cardiac troponins and CK-MB in regard to classification of stroke etiology is more limited. Associations between positive troponin levels and CE stroke have been repeatedly found during the acute phase in single cohort studies (36, 37, 75). It has also been shown that elevated troponin levels may improve the yield of abnormal echocardiography findings such as the presence of intracardiac thrombus, valvular disease, low ejection fraction, and akinetic wall segments. Therefore, troponin could be useful in selecting patients for further cardiac examinations (39, 40). However, a most recent substudy of the NAVIGATE-ESUS trial showed that high-sensitivity cardiac troponin T (hs-cTnT) was associated with increased rates of cardiovascular event but did not find any evidence supporting anticoagulation in these patients. This indicates that hs-cTnT is not useful to guide management of anticoagulation (76). Only limited evidence exists for the association of CK-MB with cardioembolism hindering any firm conclusions yet (41, 77).

## Blood-Based Biomarkers of Inflammation

Although markers of systemic inflammation such as CRP (C-reactive protein) and pro-inflammatory cytokines [e.g., Interleukin-6 (IL-6), Interleukin-1β (IL-1β), and tumor-necrosis factor-alpha (TNFα)] have been mainly evaluated in regard to the diagnosis of large-artery atherosclerosis, growing evidence suggests a prominent role of systemic inflammation in the pathophysiology of AF (78) and the prothrombotic state in AF patients (79). Thus, inflammatory biomarkers may also be helpful in the diagnosis of CE stroke. In a 2017 meta-analysis (including 14 studies, 2,751 patients), CRP was significantly higher in CE strokes compared to non-CE strokes (42). However, no difference was found between CE stroke and LAA stroke alone. A possible explanation for this might be the involvement of inflammation in the underlying pathophysiology of both etiologies that limits the accuracy of CRP as an etiological biomarker. Evidence for inflammatory cytokines (e.g., IL-6, IL-1β, and TNFα) is even more conflicting. While smaller studies have found higher levels of all three cytokines in CE stroke patients (43, 44), other studies could not find significant differences (80, 81). A 2017 meta-analysis on IL-6 (4 studies, 419 patients) could only confirm significantly higher levels in CE-stroke patients compared to lacunar strokes but not to non-CE strokes combined (42). Furthermore, positive associations were not adjusted for stroke severity; thus, higher cytokine levels in CE stroke could be also explained by larger infarcts prompting more severe immunoreaction.

## Blood-Based Biomarkers of Coagulation

D-dimer, a degradation product of cross-linked fibrin, is formed during activation of the coagulation system. In CE stroke, it is hypothesized that D-dimer levels reflect the formation of fibrin-rich thrombus in the left atrium. Contrarily, thrombi originating from large arteries are believed to be mostly platelet-rich and therefore only promote a mild D-dimer increase (82). The association of higher levels of D-dimer with CE stroke etiology when measured during the acute phase (<48 h) has been repeatedly shown in individual studies (45–50). Further evidence indicates that the discriminatory ability may increase when D-dimers are measured ultra-early (3–6 h) after symptom onset (83). Still, a positive association of D-dimer levels with CE stroke seems to remain stable beyond the acute phase (50). A major limitation of D-dimers, however, is their low specificity for a specific underlying pathophysiology. D-dimers are regularly elevated in many different thrombotic diseases but also in other systemic diseases. Some of the latter (e.g., systemic cancer, deep vein thrombosis in the presence of PFO or acute aortic dissection) are themselves associated with stroke. High D-dimer levels combined with typical DWI-lesion patterns in cryptogenic stroke patients may therefore play a role in the diagnosis of embolic stroke in the presence of occult cancer (84).

## Other Circulating Proteins

Although this review focuses on the most promising biomarkers that have proven consistent associations in multiple evaluations, it is to mention that preliminary data exists for a variety of other circulating proteins (also summarized in Table 1). However, the single-study nature of the existing evidence or conflicting results from different studies hinder any firm evaluation of their clinical usefulness yet. Further, recent advantages in proteomic studies may facilitate the discovery of novel potential biomarkers in upcoming decades.

## Genomic, Transcriptomic, and Metabolic Markers of Cardioembolic Stroke

Little research has been done on possible genetic and metabolic markers of CE stroke. However, advances in high-throughput technologies in recent decades have provided researchers with new opportunities for biomarker discovery (85). Much recently, it was demonstrated that genetic risk variants associated with AF were also associated with CE stroke when combined in a polygenetic risk score (86). Further transcriptomics studies focusing on circulating RNAs in peripheral blood of stroke patients have shown promising results (87). In a pilot study, it was shown that a minimum of 23 genes was able to differentiate CE from LAA stroke with >95% sensitivity and specificity (53). In a different study, a 40-gene profile differentiated CE stroke from large-vessel stroke with >95% sensitivity and specificity (54). A separate 37-gene profile differentiated cardioembolic stroke due to AF from non-AF causes with >90% sensitivity and specificity. Functional analysis of the genes involved highlighted a difference in the inflammatory response by various stroke subtypes. Confirmation in larger cohorts may thus inspire the development of PCR-based blood tests in the future. Only few studies have been conducted to examine whether metabolites can be used to differentiate stroke subtypes. It has been shown that levels of free fatty acids were ~1.5 fold higher in CE stroke compared to other subtypes (55). In a different study, levels of the tricarboxylic acid metabolites succinate, α-ketoglutarate, and malate were associated with CE stroke. Interestingly, all three metabolites were also associated with subclinical atrial dysfunction (56). Whether these observations may be helpful to guide secondary prophylactic treatment remains to be determined.

## Electrocardiographical Markers

Electrocardiographical (ECG) markers may similarly reveal subclinical cardiac pathologies. For etiological classification, possible markers focus mainly on abnormalities of the cardiac atria, as the left atrium is considered the main source of thrombus formation both, in the presence and absence of AF. P-wave terminal force in lead V1 (PTFV1) is an established marker of left atrial abnormality and was found to be an independent predictor of incident ischemic stroke as well as incident AF in meta-analysis (88, 89). While small studies have failed to provide direct evidence that PTFV1 can distinguish between etiologies in adult stroke patients (90), an association of PTFV1 with CE-stroke was seen in the Helsinki Young Stroke Registry reporting on ischemic strokes in patients aged 15 to 49 years (91). Further, indirect evidence from longitudinal-cohort studies supports an association with CE stroke etiology in adults: In a case–cohort analysis of the Northern Manhattan Study, the association of PTFV1 with the incidence of stroke was limited to CE and undetermined stroke. No association was found with non-CE stroke subtypes (57). Similarly, the atherosclerosis risk in communities study could demonstrate an association with non-lacunar but not with lacunar strokes (58). In both studies, the association was independent of the presence of AF; thus, PTFV1 may be a marker identifying underlying atrial myopathy as it is linked to atrial fibrosis. Further, in patients with embolic stroke of undetermined source (ESUS), increased PTFV1 was inversely associated with unstable extracranial sub-stenotic atherosclerosis as a potential cause of unrecognized LAA-associated stroke (92). Conflicting evidence exists in regard to the predictive value of PTFV1 for newly diagnosed AF after stroke (93, 94). Another possible ECG marker of cardiac embolism is the presence of interatrial block (IAB) or excessive supraventricular ectopic activity (ESVEA). In a retrospective single-center study, IAB has been significantly more prevalent in CE stroke compared to non-CE stroke. Interestingly, IAB was also found to be associated with CE stroke in patients with sinus rhythm (59). In ESUS patients, IAB was associated with the diagnosis of subclinical AF and stroke recurrence (95, 96). ESVEA [defined as 30 premature atrial contractions (PACs) per hour daily, or any runs of ≥20 PACs] was found to be associated with an increased risk of stroke in a long-term follow-up, independent of AF (97, 98). In a different study, the association of PACs with incidence stroke was limited to non-lacunar stroke (99). Further retrospective data showed that the number of PACs was higher in CE stroke and cryptogenic stroke compared to non-cardioembolic stroke (60).

In conclusion, preliminary evidence strongly supports a possible role of ECG parameters for etiological stroke classification. However, further studies are required to confirm this association and unravel possible clinical implications.

## Markers From Echocardiography and Advanced Cardiac Imaging

Transthoracic and transesophageal echocardiography, cardiovascular computer tomography (cvCT), and cardiac magnetic resonance imaging (CMR) may reveal previously undetected sources of cardioembolism during stroke workup, e.g., LA thrombus, ventricular thrombus, intracardial masses, aortic arch atheroma, and PFO (100, 101). Despite that, cardiac imaging is useful to provide volumetric and functional assessment of the LA. LA volume index (LAVI) measurements by echocardiography have been shown to be associated with CE-stroke etiology (61–63). In line with that, moderate to severe LA enlargement, assessed by LA diameter, has also been found to be associated with recurrent cardioembolic/cryptogenic stroke risk, but not with total recurrent stroke risk in the Northern Manhattan Stroke Study (102). Most recently, another study was able to demonstrate that left atrial diameter above 40 mm, may be useful to select patients for PCM (65). Furthermore, LA diameter was also associated with stroke recurrence in AF patients (103). Indirect evidence of a beneficial effect of oral anticoagulation was obtained in a secondary analysis of the NAVIGATE-ESUS trial: A subgroup of patients with moderate or severe LA enlargement treated with rivaroxaban showed a reduced risk of recurrent stroke (64). More recently, speckle tracking echocardiography-derived evaluation of LA deformation (LA strain) has gained attention as a possible novel marker of LA dysfunctionality (104). In a pilot study, low LA strain was associated with higher detection rates of paroxysmal AF in cryptogenic stroke patients (66). This association has been confirmed by a different study when measures of LA strain were combined with NT-proBNP (105). Further, measures of LA strain were shown to be independently associated with stroke occurrence in AF patients (106). Therefore, LA measurements might be another promising tool contributing to management of stroke preventive strategies.

The gold standard for the evaluation of LA enlargement remains CMR as it allows for 3D reconstruction as well LA hemodynamics (107, 108). Delayed-enhancement CMR (DE-CMR) provides a noninvasive way to characterize and quantify LA fibrosis in the general population, including individuals without known structural heart disease or AF (109). Retrospective multicenter studies including patients with AF have been able to show that DE-CMR was independently associated with a history of stroke (110) and future cerebrovascular events during follow-up (111). Patients with undetermined stroke had similar rates of LA fibrosis compared to patients with CE stroke, but higher rates compared to patients with other specific causes (67). This suggests presence of subclinical atrial disease in those patients. Comparable results were seen in ESUS patients. They showed similar rates of LA fibrosis compared to AF patients, but higher rates compared to healthy controls (112). Thus, LA fibrosis assessed by CMR could also be a promising marker for identifying underlying atrial myopathy and corresponding cardioembolic stroke risk in the future. However, further prospective studies need to investigate if DE-CMR is associated with risk of stroke in patients without AF before clinical trials may evaluate a benefit of anticoagulation in this patient population.

## Future Perspectives

Since the failure of the NAVIGATE-ESUS and RESPECT-ESUS trials to demonstrate a benefit of oral anticoagulation in patients with ESUS, optimal secondary prevention still remains elusive in a significant amount of patients. A more rigorous etiological workup may lead to the identification of more patients with competing etiological factors [like it has been seen in the NAVIGATE ESUS trial (113)]. Thus, it became obvious that optimal treatment has to be even more suited to individualized patient risk in regard to future cerebrovascular events.

Biomarkers add relevant information for this endeavor, specifically CE biomarkers alone or incorporated into validated risk scores, could guide clinicians selecting patients for more excessive workup (e.g., PCM). Examples for existing risk scores are the STAF, iPAB, and ASAS scores (114–116). They incorporate levels of BNP or presence of LA enlargement into clinical scores to predict AF after cerebrovascular events. This may increase the yield of positive PCM findings. Yet, external validation and cost effectiveness studies are pending. These studies are crucial to define adequate risk thresholds for patient selection. More importantly, biomarkers could have the potential to guide decision over secondary prevention strategies as indirect evidence already suggests. However, to pave the road to clinical application, the inherent shortcomings of etiological classification systems need to be overcome by the conduction of well-designed randomized trials.

Three trials that incorporate biomarkers to define target populations are currently enrolling: In the US, The Atrial Cardiopathy and Antithrombotic Drugs in Prevention After Cryptogenic Stroke (ARCADIA; NCT03192215) randomized trial evaluates apixaban compared to aspirin in ESUS patients with presumed atrial myopathy (defined by NTproANP levels > 250 pg/ml, PTFV1 > 5000V x ms or LA enlargement defined as LA diameter index ≥ 3 cm/m^2^ on echocardiography). In Europe, the Midregional Proatrial Natriuretic Peptide to guide Secondary Stroke Prevention (MOSES; NCT03961334) randomized trial evaluates oral anticoagulation vs. antiplatelet therapy in patients without previous AF (selected by a MRproANP levels ≥ 200 pg/l) and the Apixaban for Treatment of Embolic Stroke of Undetermined Source (ATTICUS; NCT02427126) trial is evaluating apixaban compared to aspirin in ESUS patients with at least one non-major risk factor for cardioembolism including possible echo- and electrocardiography markers such as LA size >45 mm in parasternal axis, reduced LAA flow velocity ≤0.2 m/s, spontaneous LAA contrast, atrial high rate episodes, and PFO. If completed successfully, data from these trials not only could provide compelling evidence for the concept of atrial myopathy but also may prove the benefit of biomarker-guided therapy.

Thus, biomarkers of CE-stroke etiology could ultimately evolve as a cornerstone in decision making regarding secondary stroke prevention.

## Author Contributions

AM wrote the first draft of the manuscript. SV and KM wrote sections of the manuscript. All authors contributed to manuscript revision, read, and approved the submitted version.

## Conflict of Interest

The authors declare that the research was conducted in the absence of any commercial or financial relationships that could be construed as a potential conflict of interest.
